# Characterization of Intrinsically Disordered Prostate Associated Gene (PAGE5) at Single Residue Resolution by NMR Spectroscopy

**DOI:** 10.1371/journal.pone.0026633

**Published:** 2011-11-02

**Authors:** Maarit Hellman, Helena Tossavainen, Pekka Rappu, Jyrki Heino, Perttu Permi

**Affiliations:** 1 Program in Structural Biology and Biophysics, Institute of Biotechnology, University of Helsinki, Helsinki, Finland; 2 Department of Biochemistry and Food Chemistry, University of Turku, Turku, Finland; University of South Florida College of Medicine, United States of America

## Abstract

**Background:**

The Cancer-Testis antigens (CTA) are proteins expressed in human germ line and certain cancer cells. CTAs form a large gene family, representing 10% of X-chromosomal genes. They have high potential for cancer-specific immunotherapy. However, their biological functions are currently unknown. Prostate associated genes (PAGE) are characterized as CTAs. PAGE5 is one of six proteins belonging to this protein family, also called CT16.

**Methodology/Principal findings:**

In this study we show, using bioinformatics, chromatographic and solution state NMR spectroscopic methods, that PAGE5 is an intrinsically disordered protein (IDP).

**Conclusion/Significance:**

The study stands out as the first time structural characterization of the PAGE family protein and introduces how solution state NMR spectroscopy can be effectively utilized for identification of molecular recognition regions (MoRF) in IDPs, known often as transiently populated secondary structures.

## Introduction

The Cancer-Testis antigens (CTAs) are expressed mainly in normal human trophoblasts and germ line i.e. testis and placenta, but not in other healthy cells [1]. Some cancer cells turn on CTA expression by epigenetic regulation, i.e. by DNA hypomethylation and histone post-translational modifications [2]. Majority of CTA genes are X-chromosome linked and CTAs represent 10% (99 in total) of all X-chromosomal genes [3]. These genes include GAGE, MAGE, SSX, NXF, SPANX, CSAGE, ESO/LAGE gene families, which have been found by X-chromosome DNA sequencing and sequence analysis [3]. Expression profile of X-chromosome linked CTAs is more restricted when compared to non-X-linked ones [4]. Limited expression profile and readily recognizable target for cancer patient immune system render CTAs highly useful for cancer-specific immunotherapy i.e. having great potential as therapeutic cancer vaccines in specific cancer [5], [6], [7], [8], [9]. Recently, publicly available knowledge-based database of CTAs has been established (http://www.cta.lncc.br/index.php) due to the increasing interest towards CTAs and their applications [10]. Prostate associated genes (PAGEs) together with their sequentially homologous proteins, X antigens (XAGEs) and G antigens (GAGEs), are members of GAGE gene family products [11]. Exact biological functions of these proteins, either in prostate or cancer, remain to be characterized, although recent studies have highlighted anti-apoptotic properties for PAGE4 [12] and GAGE7 [13]. Interestingly, cancer cell resistance to chemo- and radio-therapies, has been associated to the anti-apoptotic features of GAGE7 [13]. There are six different PAGE proteins (PAGE1, 2, 2B, 3, 4, and 5) [11], [14], [15], expressed in prostate or testis and also in several cancer cells. PAGE5 has been recognized as potential marker for diagnosis of specific cancers as increased expression levels are observed in melanoma, renal and lung cancer cells [4], [16], [17].

Members of PAGE family are small proteins containing 102–146 amino acids. A more careful examination of amino acid composition reveals high abundance of charged/hydrophilic residues and few hydrophobic residues, characteristic for intrinsically disordered proteins (IDPs) [18], [19]. Very recently, using bioinformatics tools together with CD and ^1^H NMR spectroscopy, PAGE4 has been characterized as a disordered protein that contains an N-terminal nuclear localization signal (NLS). In addition, biochemical assay showed that PAGE4 binds dsDNA [12]. However, more detailed structural studies are needed of GAGE gene family products.

During past several years, increasing number of studies regarding IDPs and proteins with disordered regions (IDRs) has been reported, thus increasing our knowledge (and awareness) of proteins that lack well-defined three-dimensional structure but which exhibit essential biological function, thus challenging the structure defines function paradigm. In addition to classical, rigid lock-key binding model established for many folded proteins, enzyme dynamics in terms of conformational selection or induced fit is general feature of protein interactions and interaction of disordered protein with a ligand may induce (partial) folding for unstructured parts [20]. However, a protein-protein interaction mode does not necessitate folding and it may take place without well-ordered conformations, a property termed as fuzziness [21]. IDPs and IDRs cannot necessarily be described as random flight chains but often contain short recognition sites such as preformed structural elements (PSE) [22], molecular recognition regions (MoRFs) and eukaryotic linear motifs (ELMs). PSEs are short disordered regions in IDPs, which have tendency for formation of transiently populated secondary structures, which may function as potential ligand binding sites [22]. MoRFs are short segments in protein, which upon binding to their ligands undergo disorder-to-order transitions [23]. In addition, ELMs use distinct mechanisms exhibiting disordered recognition sites of proteins with exposed regions with characteristic physicochemical properties [24]. Disorder-to-order transition upon binding is thermodynamically unfavorable. In folded proteins, the bound conformation may already exist whereas in IDPs the disordered binding region folds into a binding conformation, resulting in entropic penalty to Gibbs free energy of binding. However, disorder-to-order transition offers several functional benefits: low affinity and reversible binding, fast ligand binding and ability to bind several ligands (moonlighting) [25]. Furthermore, it enables dissecting of affinity from specificity enabling highly specific interactions with low affinity. Consequently, IDPs are often involved in regulatory processes and signaling. From cellular compartments, nucleus is most enriched with IDPs or IDRs [18].

In this work, we have employed bioinformatics, chromatographic methods as well as solution state NMR spectroscopy for structural and functional characterization of PAGE5. We show that PAGE5 is structurally disordered protein but contains transiently populated structural elements. We also show that the elements are more populated at lower pH. In addition, our preliminary studies revealed no binding with double stranded DNA similar to PAGE4. The present study introduces for the first time the structural and dynamic characterization of GAGE gene family proteins at single residue resolution.

## Results and Discussion

### Size exclusion chromatography and bio-informatics prediction of PAGE5

**Figure 1:**
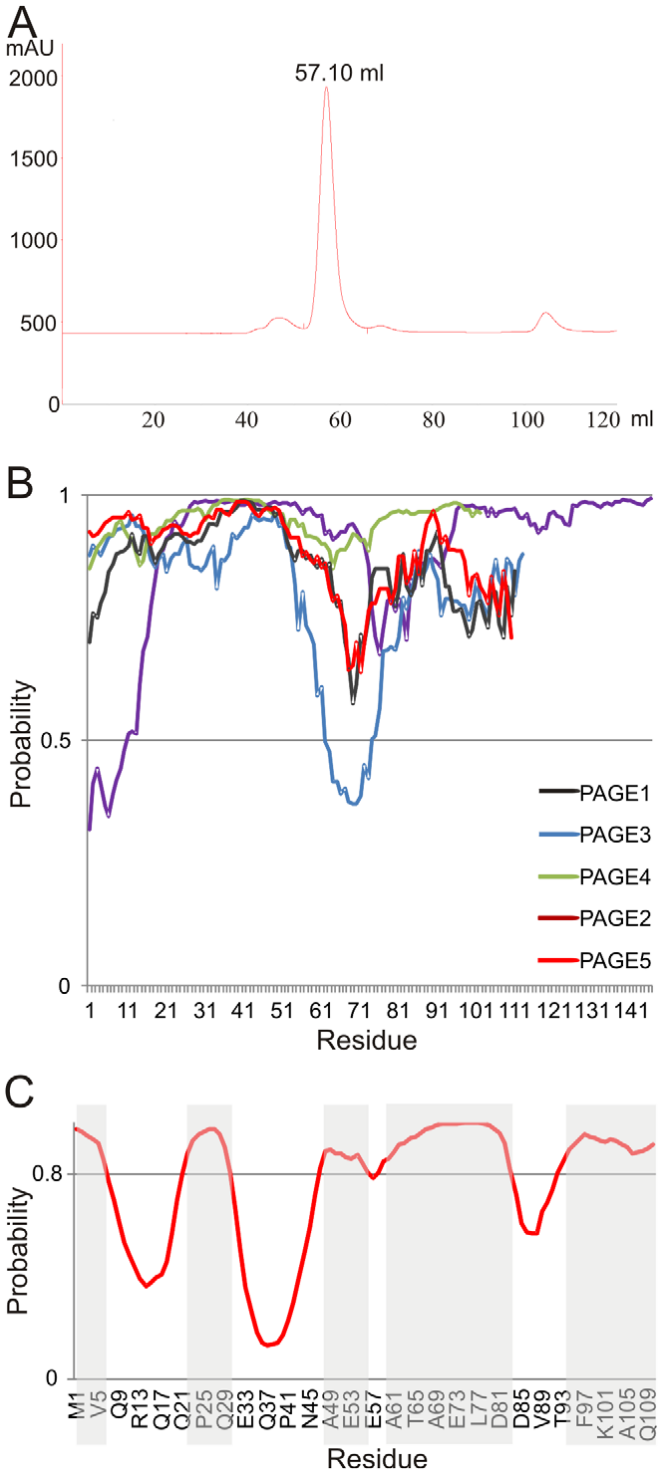
Size exclusion chromatogram of PAGE5 and IUPred analysis of PAGE proteins. (**A**) PAGE5 elutes as a single peak from Superdex S75 (16/60) column, suggesting molecular weight of 44 kDa, which is approximately four times higher than actual molecular mass, 11.8 kDa. Void volume of the column was determined experimentally to 39 ml. Column was calibrated by using ovalbumin (elution volume = 58 ml, MW = 43 kDa) and chymotrypsinogen (elution volume = 68 ml, MW = 25 kDa) as a standard proteins (GE Healthcare). (**B**) IUPred Software [47] prediction suggests that all PAGE family proteins are highly disordered. (**C**) MoRFs of PAGE5 predicted by ANCHOR Software [48]. Residues forming the MoRFs with propability larger than 80% are shaded.

The PAGE5 protein is a highly soluble protein at high concentrations (1.5 mM). According to size exclusion chromatogram (SEC), the last step of the purification procedure, PAGE5 migrates with volume characteristic for globular protein with molecular weight of 44 kDa (Figure 1A)). Since molecular weight of monomeric PAGE5 is only 11 kDa, SEC gave an estimation of four times larger MW, i.e. tetrameric protein. As protein migration at SEC column is affected in addition of molecular weight also by shape of the protein, we studied structural features of PAGE5 further by means of NMR spectroscopy. We also used IUPred software for the prediction of unstructured parts of PAGE5 and compared it with other PAGE family proteins (Figure 1B). According to the prediction all PAGE proteins are highly disordered. PAGE3 shows the only exception at the region of residues 62–73. Residues are characterized as disordered, if the disorder tendency (DT) exceeds 0.5 (Figure 1B). Exceptions are residues 62–73 of PAGE3, where DTs are between 0.37 and 0.49. Corresponding residues for PAGE5 and PAGE2 show lower than average DT (<0.7) for residues 68–72. Also N-terminal residues 1–10 of PAGE1 are predicted to form structural region, showing DT between 0.32 and 0.48. From other PAGE family proteins only PAGE2's N-terminus has lower DTs, i.e 0.70–0.77 for residues 1–4. According to IUPred prediction, PAGE4 has the most disordered structure, shown by the highest average disorder tendency (>0.94). Regions with lowered DT can be predicted as MoRFs and might explain the different appearances in distinct cancer types. ANCHOR software was used for prediction of MoRFs (Figure 1C). We also used PSIPRED server for predicting secondary structure elements in PAGE5. PSIPRED analysis suggests α-helical segment for residues 67–79 in PAGE5 (data not shown).

### Assignment of NMR resonances in PAGE5

**Figure 2:**
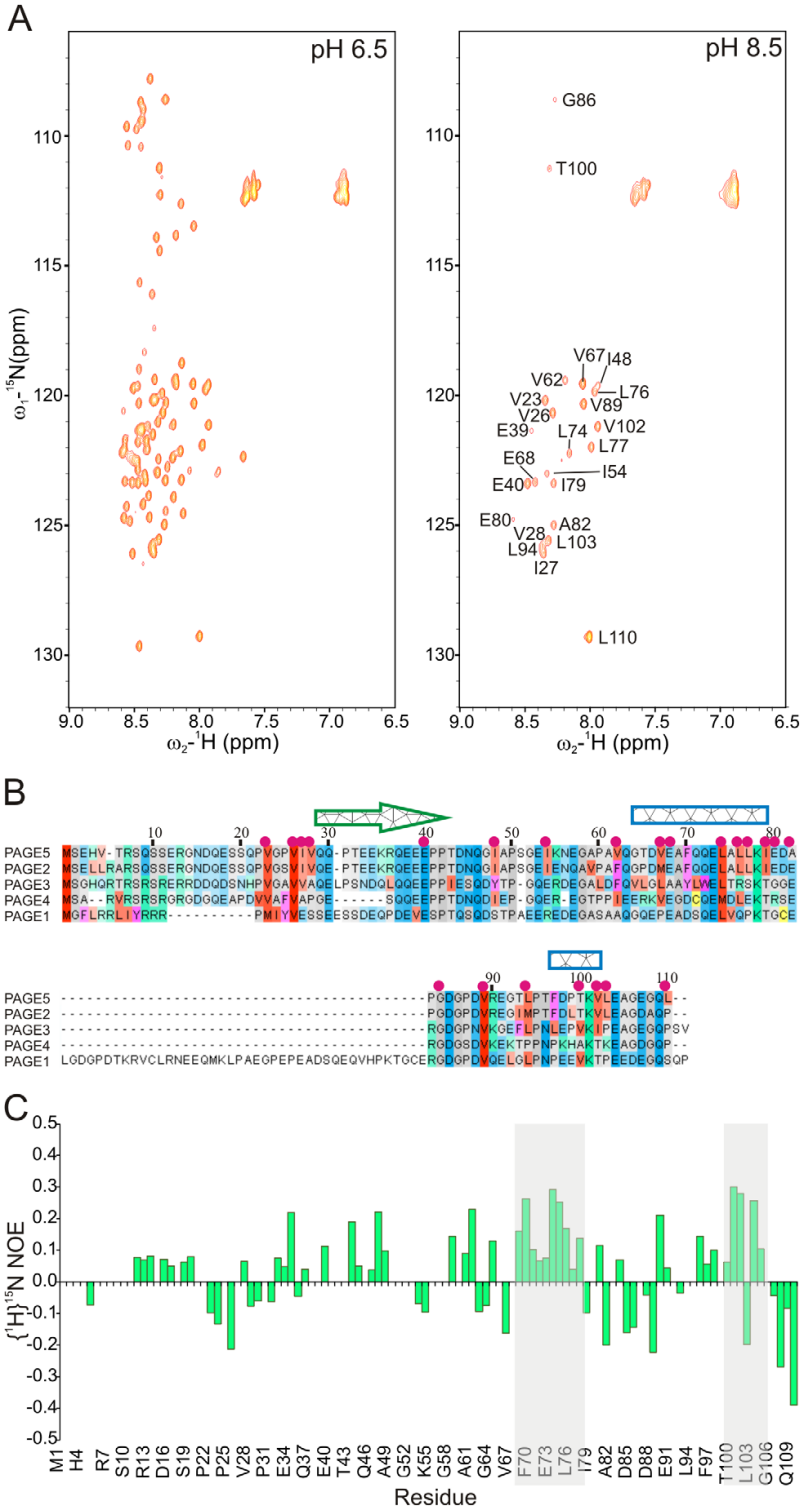
^15^N-HSQC spectra and heteronuclear NOE suggest PAGE5 as IDP. (**A**) 2D ^15^N-HSQC spectra of uniformly ^15^N ^13^C labelled PAGE5, recorded at pH 6.5 and 8.5. Assignments for remaining 23 HN signals at high pH are labeled into the spectrum. Visible correlations belong mostly to hydrophobic amino acids, also located at the region of possible PSE. (**B**) Sequence alignments of proteins belonging to PAGE family. Correlation peaks, which remained visible at high pH (8) are labelled above the sequence with magenta spheres. Suggested transient alpha helical and beta structures are marked with rectangular and arrow, respectively. (**C**) Steady-state {^1^H}-^15^N heteronuclear NOE values as a function of amino acid sequence. Regions with suggested transient secondary structure elements are shaded.

Next we employed NMR spectroscopy to characterize the structure and dynamics of PAGE5 in solution. Figure 2A shows a two-dimensional ^15^N, ^1^H correlation spectrum (^15^N-Heteronuclear Single Quantum Coherence) of ^15^N, ^13^C labeled PAGE5. The spectrum displays poorly dispersed ^15^N, ^1^H correlations, a hallmark of a disordered protein, stemming from highly similar chemical environment of amide protons due to rapid interconversion of conformers. Further inspection of aliphatic proton chemical shifts, especially lack of dispersion in the methyl proton region, supports the initial observations made on amide proton chemical shifts i.e. underscoring the disordered nature of PAGE5 (data not shown). At pH 8.5, only 23 amide correlation peaks remained detectable, indicating accelerated amide proton exchange with water, where amide protons are not protected by the globular structure (Figure 2A and 2B). Further evidence of the disordered PAGE5 was obtained by measuring steady-state {^1^H}-^15^N heteronuclear NOEs, which report rigidity of the protein backbone (Figure 2C). For residues associated to secondary structure elements in rigid molecules, heteronuclear {^1^H}-^15^N NOEs have typically values larger than >0.7. In case of highly disorderd protein backbone, hetNOEs display negative values or values very close to zero. The HetNOE plot as a function of amino acid sequence of PAGE5 shows small positive and negative NOEs with several zero crossings, pinpointing the disordered nature of PAGE5 backbone. However, some amino acid segments exhibit clearly positive hetNOEs indicating existing transient structural rigidity in PAGE5 (*vide infra*).

The number of emerging correlations in the ^15^N-HSQC spectrum indicated absence of few ^15^N, ^1^H cross peaks owing to linebroadening stemming from µs-ms timescale dynamics or increased NH exchange rate with solvent (*vide infra*). However, the chemical shift assignment was initially made using iHNCACB [26] and CBCA(CO)NH [27] experiments, which turned out to be an unsuccessful strategy for PAGE5 despite highly selective intraresidual and sequential magnetization transfer schemes utilized in these experiments, respectively. As ^13^C′ chemical shifts in IDPs are typically less clustered in comparison to ^13^Cα/^13^Cβ shift [28], the ^13^C′ chemical shift-based assignment approach was next employed using i(HCA)CONH [29] and HNCO [30] experiments that provide solely intraresidual ^1^H(*i*), ^15^N(*i*) and ^13^C′(*i*) and sequential ^1^H(*i*), ^15^N(*i*) and ^13^C′(*i*-1) correlations, respectively. In this way, a nearly complete assignment of ^1^H^N^, ^15^N, ^13^C′, ^13^Cα and ^13^Cβ resonances was obtained. However, one proline residue as well as the N-terminal segments ^1^MSEH^4^ and ^8^SQSS^11^ remained unassigned. We reckoned that the absence of NH correlations in the N-terminal part is due to rapidly exchanging amide protons and to extend resonance assignments for these residues, we employed a suite of Hα-detected experiments that are less susceptible to fast NH exchange rates [31], [32]. Using this approach, we were able to obtain a nearly complete assignment of ^1^Hα, ^13^C′, ^13^Cα and ^15^N resonances also in the N-terminal part of PAGE5 (Supplementary Table S1).

### Chemical shift analysis reveals transiently populated secondary structure elements

**Figure 3:**
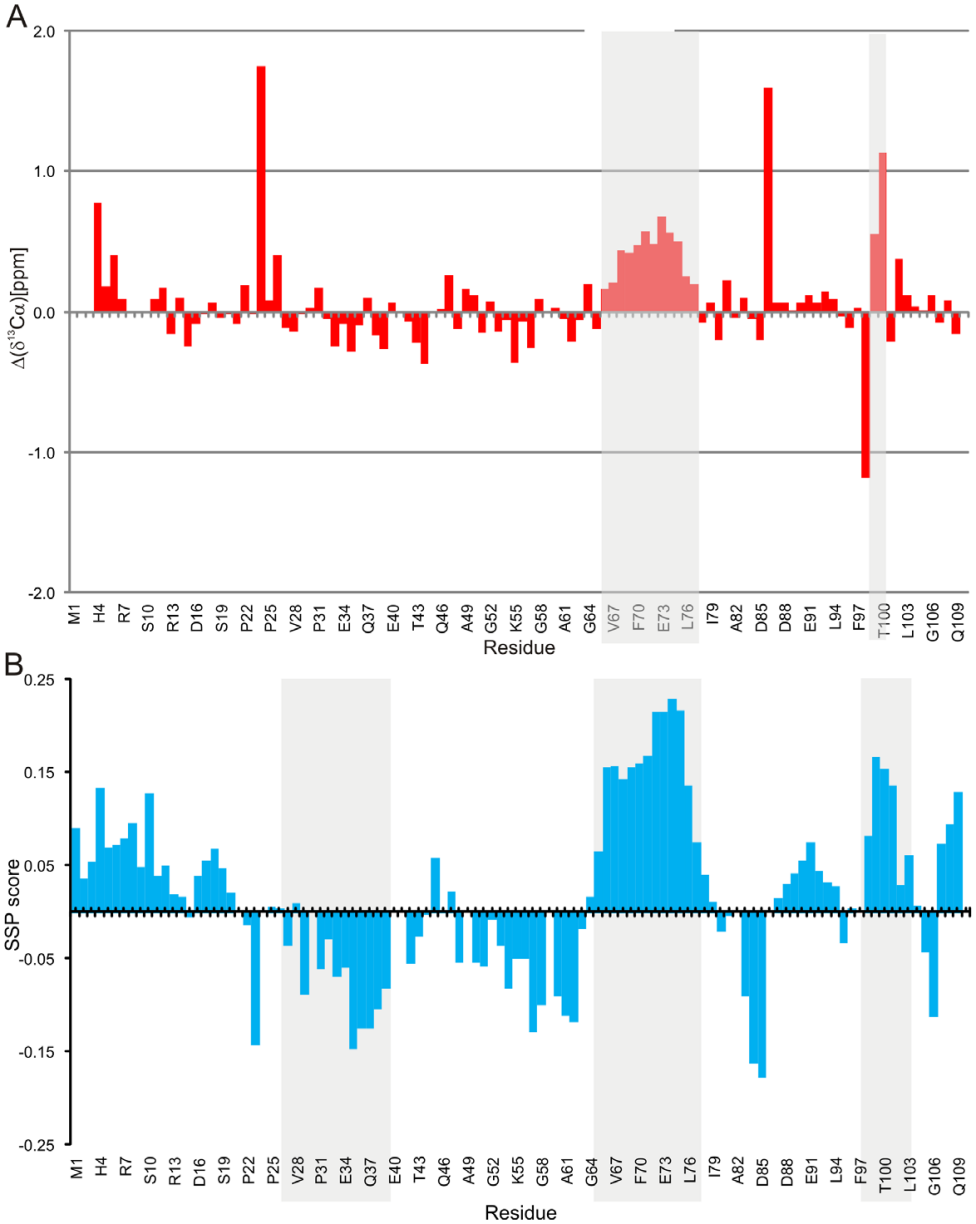
Secondary structure prediction from ^1^Hα, ^13^Cα, ^13^Cβ chemical shift of PAGE5. (**A**) Chemical shift deviations from random coil shifts for ^13^Cα(red bars) as a function of primary structure. (**B**) Secondary structure propensity score for PAGE5. ^1^Hα, ^13^Cα, ^13^Cβ chemical shifts were used for calculations. Regions with suggested transient secondary structure elements are shaded.

NMR chemical shifts are extremely sensitive reporters of transient structural motifs. In proteins, so-called secondary chemical shifts can be used for probing fractional secondary structure e.g. transient α-helices or extended conformations [33]. We compared the nearest neighbor effect corrected random coil chemical shifts obtained from Ac-QQXQQ-NH2 peptide recorded at neutral pH and milder urea concentration [34] to experimentally observed chemical shifts of PAGE5 [34]. A positive (negative) inclination of ^13^Cα and ^13^C′ chemical shifts from the corresponding random coil shifts is an indication of α-helical (β-structure) propensity for a given segment of residues. A similar but opposite phenomena can be observed for ^15^N chemical shifts i.e. chemical shifts that are negative (positive) indicate propensity for α-helical (β-structure) conformation. Figure 3A shows secondary chemical shifts for ^13^Cα spins as a function of amino acid sequence of PAGE5. Chemical shift data reveal that PAGE5 is mostly disordered protein but it contains a few transiently populated secondary structure elements or local structural segments. ^13^Cα shifts are the most reliable indicator of any residual secondary structure and clearly highlight consecutive positive secondary chemical shifts for a region encompassing residues ^66^Asp-Val-Glu-Ala-Phe-Gln-Gln-Glu-Leu-Ala-Leu-Leu^77^. This strongly suggests presence of fractional α-helical conformation in this region. These observations coincide closely with ^13^C′ chemical shift data that display significant positive deviation from random coil shifts of residues in ^65^Thr-Asp-Val-Glu-Ala-Phe-Gln-Gln-Glu-Leu-Ala^75^ indicating that the polypeptide have a bias to α-helical and β-strand (extenteded) conformations (not shown). In addition, large deviations from random coil shifts for residues in the C-terminal segment ^99^-Pro-Thr^100^ hints nascent local structural order for this short stretch. The region encompassing residues ^32^Thr-Glu-Glu-Lys-Arg-Gln-Glu-Glu-Glu-Pro-Pro^42^ shows much vaguer tendency to negative ^13^Cα (as well as ^13^C′, not shown) secondary chemical shifts, which makes the observation of more extended conformation elusive. Next, a more quantitative analysis is given by the secondary structure propensity (SSP) score [35] using ^1^Hα, ^13^Cα, ^13^Cβ chemical shifts, was employed. In the SSP analysis, α-helical and extended (β-strand) structures will get positive and negative scores, where +1 and −1 indicate fully formed α-helix or β-structure, respectively. For PAGE5, the regions encompassing residues ^65^Thr-Asp-Val-Glu-Ala-Phe-Gln-Gln-Glu-Leu-Ala-Leu-Leu^77^ and ^29^Gln-Gln-Pro-Thr-Glu-Glu-Lys-Arg-Gln-Glu-Glu-Glu-Pro-Pro^42^ populate α-helical and extended conformations albeit the corresponding propensities are low 18% and 9%, respectively (Figure 3B). While this is in good accordance with analysis based on ^13^Cα (and ^13^C′) secondary chemical shifts for helical segments some discrepancy exist in determining the extended structures. To conclude, NMR chemical shift data correlates well with the secondary structure prediction made by PSIPRED algorithm which suggested propensity for α-helical conformation in residues ^67^ Val-Glu-Ala-Phe-Gln-Gln-Glu-Leu-Ala-Leu-Leu-Lys-Ile^79^.

### Heteronuclear ^2^J_NCα_ and ^1^J_NCα_ couplings reveal tendency towards transient secondary structure

**Figure 4:**
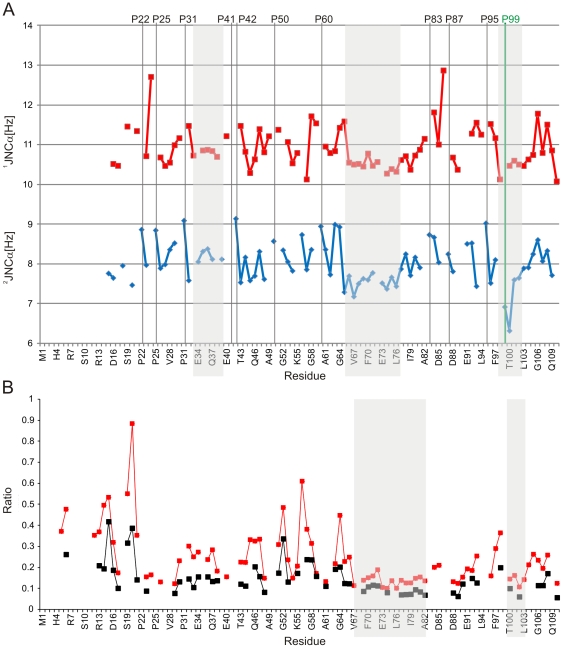
One and two bond NCα scalar J couplings of the PAGE5 and HN exchange with water. (**A**) Plot of ^1^J(NCα) and ^2^J(NCα) couplings in Hertz, with red and blue lines, respectively. (**B**) Plot of ratio of the CLEANEX experiments and reference 2D ^15^N-HSQC spectrum with 25 ms (red) and 10 ms (black) mixing times as a function of amino acid sequence. Regions with suggested transient secondary structure elements are shaded.

Although secondary chemical shifts are highly useful for identification of transiently populated secondary structure elements in IDPs, further evidence can be obtained from analysis of *J* couplings. Given that observed scalar couplings are population weighted averages of couplings sampled over various conformations, any deviation from random coil values can be interpreted as a secondary coupling contribution in analogy to secondary chemical shifts. While quantitative description of the relation between protein secondary structure and one-bond couplings between ^15^N(*i*) and ^13^Cα(*i*) (^1^J_NCα_) or two-bond couplings between ^15^N(*i*) and ^13^Cα(*i*-1) (^2^J_NCα_) is difficult, ^2^J_NCα_ is extremely valuable in distinguishing between α-helical or turns, and β-structure [36]. Indeed, fully formed α-helix exhibits ^2^J_NCα_ couplings varying within the range 5.5–7 Hz, whereas β-structures display ^2^J_NCα_ couplings between 8–10 Hz [36], [37]. Likewise, ^1^J_NCα_ couplings larger than 11 Hz can be associated to β-strands i.e. ψ angles 120–180°, whereas values smaller than 9.5 Hz are typically not found for β-strands (ψ∼100–180°). Observed ^2^J_NCα_ couplings for the ^64^Gly-Thr-Asp-Val-Glu-Ala-Phe-Gln-Gln-Glu-Leu-Ala-Leu-Leu^77^ segment show a consecutive stretch of smaller than average values in comparison to flanking regions, which is in good accordance with the transiently populated α-helix recognized in the secondary chemical shift analysis above (Figure 4A). Likewise, ^1^J_NCα_ couplings show diminished values for this part of the PAGE5 sequence, providing further evidence of fractional α-helicity. In contrast, residues ^32^Thr-Glu-Glu-Lys-Arg-Gln-Glu^38^ which according to the SSP analysis populate β-strand for a given fraction of time, show slightly elevated values for ^2^J_NCα_ coupling, which supports observation based on secondary chemical shifts i.e. transient extended conformation found for this region. Pro-31 in the middle of the segment is likely to induce a kink to a β-strand. Interestingly, residues ^97^Phe-Asp-Pro-Thr-Lys-Val^102^ also display small ^1^J_NCα_ or ^2^J_NCα_ couplings, which fit in with perception of a short helical stretch in the SSP score analysis. It is noteworthy that prolines have significantly larger ^2^J_NCα_ couplings than the vast majority of non-proline residues. However, Pro-99, which is located in the ^97^Phe-Asp-Pro-Thr-Lys-Val^102^ motif has drastically smaller ^2^J_NCα_ coupling value, further supporting local structural ordering for this segment (Supplementary Table S1).

### Transiently populated secondary structures show decreased exchange rates with solvent

Labile amide protons that are part of rigid, structured segments in the amino acid sequence are typically protected from chemical exchange process with solvent. In contrast, residues in flexible parts of the polypeptide chain have typically solvent exposed amide protons with modest protection against solvent exchange i.e. they show increased exchange rates compared to residues that are part of secondary structures. This exchange phenomenon can be studied using H/D spectroscopy, where site-specific signal decay is monitored after dissolving the protein sample into D_2_O. In case of IDPs, this is often impractical as H/D exchange is relatively rapid in comparison to globular proteins. Instead, selective saturation transfer from solvent protons to amide protons using the so-called CLEANEX-PM experiment [38] can be employed. In this approach, water magnetization is selectively transferred to amide protons in a series of spectra with increasing mixing times. Figure 4B shows observed ratio of saturated vs. reference spectra of amide proton cross peak intensities for two mixing times (10 ms and 25 ms). Those residues, which are less accessible to solvent show decreased ratios compared to solvent exposed residues especially with shorter mixing times. Strikingly, the C-terminal part of PAGE5, especially residues ^69^Ala-Phe-Gln-Gln-Glu-Leu-Ala-Leu-Leu^77^ and ^99^Pro-Thr-Lys-Val^102^, exhibit significant protection from solvent exchange, indicating presence of local structural motifs in these regions. In contrast, the N-terminal part of PAGE5 is clearly more prone to exchange with solvent.

### Reduced spectral density mapping indicates restricted sub-nanosecond motions in regions with fractional ordering

It is evident that internal molecular dynamics deviate between fully formed secondary structure elements and random flight chain due to more restricted motional freedom in the former. NMR spectroscopy offers unique opportunity to study protein dynamics at residue-level by measuring ^15^N auto-correlated relaxation rates [39]. Therefore, observed variation in local dynamics reports differences in molecular motions in these areas, which in turn is an indication of difference in local rigidity or stiffness of polypeptide backbone.

Three different ^15^N relaxation rates can readily be measured using a ^15^N labeled sample. Steady-state heteronuclear {^1^H}-^15^N NOEs, ^15^N longitudinal (*R*_1_) and ^15^N transverse (*R*_2_) rates, expressed in terms of the spectral density function, *J*(ω), for dipolar relaxation of ^15^N by ^1^H spin are defined as
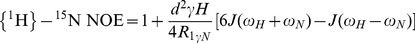
(1)

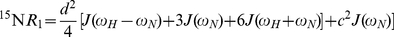
(2)

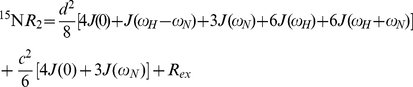
(3)where 
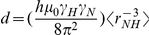
 and 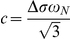, ω_H_ and ω_N_ are the Larmor frequencies of ^1^H and ^15^N, γ_H_ and γ_N_ are the gyromagnetic ratios of ^1^H and ^15^N, *h* is Planck's constant, μ_0_ is the permeability of free space, r_NH_ corresponds to the N-H bond length (1.02 Å) and Δσ is the chemical shielding anisotropy with an axially symmetric tensor (Δσω_N_ = −160 ppm). *R*_ex_ corresponds to the chemical exchange term, which adds to observed *R*_2_ rates, if present.

As can be inferred from Eqs. 1–3, {^1^H}-^15^N NOEs are sensitive to high frequency backbone motions undergoing in picosecond timescales, whereas ^15^N longitudinal (*R*_1_) and transversal (*R*_2_) relaxation rates are sensitive to motions taking place in slower ps-ns timescales. In addition, ^15^N *R*_2_ relaxation rates include plausible contribution of slower motions occurring in µs-ms timescales due to conformational exchange. Hence, analysis of ^15^N relaxation rates enables dissection of backbone dynamics in ps-ms timescales.

Classical model-free analysis [40] applied to globular proteins, where overall rotational correlation time (τ_c_) and fast internal, site-specific, motions (τ_e_) are distinguished from each other is not an appropriate description of dynamics in IDPs as deconvolution of fast internal dynamics from overall molecular tumbling is violated. A more useful approach is the so-called reduced spectral density mapping (RSDM) [39], [41], [42] that describes spectral densities in three different frequencies, *J*(0), *J*(ω_N_) and *J*(0.87ω_H_). In this approach, given that γ_N_/γ_H_ = 0.101, justified simplification is made by assuming *J*(ω_H_±ω_N_)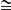 *J*(ω_H_) and Eqs. 1–3 now become
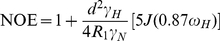
(4)

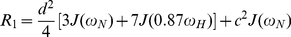
(5)

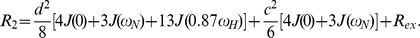
(6)It is now possible to derive values of *J*(0), *J*(ω_N_) and *J*(0.87ω_H_) from Eqs. 7–9
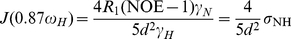
(7)

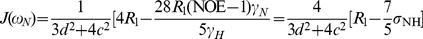
(8)

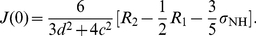
(9)
*J*(0), which is related to both ^15^N *R*_2_ and *R*_1_ maps spectral densities in ps-ns timescales but contains also contribution from slower µs-ms timescales that is mainly governed by conformational exchange (*R*_ex_ in Eq. 3). In contrast, *J*(0.87ω_H_) is only sensitive to motions on-going in subnanosecond timescales, whereas *J*(ω_N_) is sensitive to ps-ns timescales although faster (ps) and slower (ns) motions cannot be readily discriminated.

**Figure 5:**
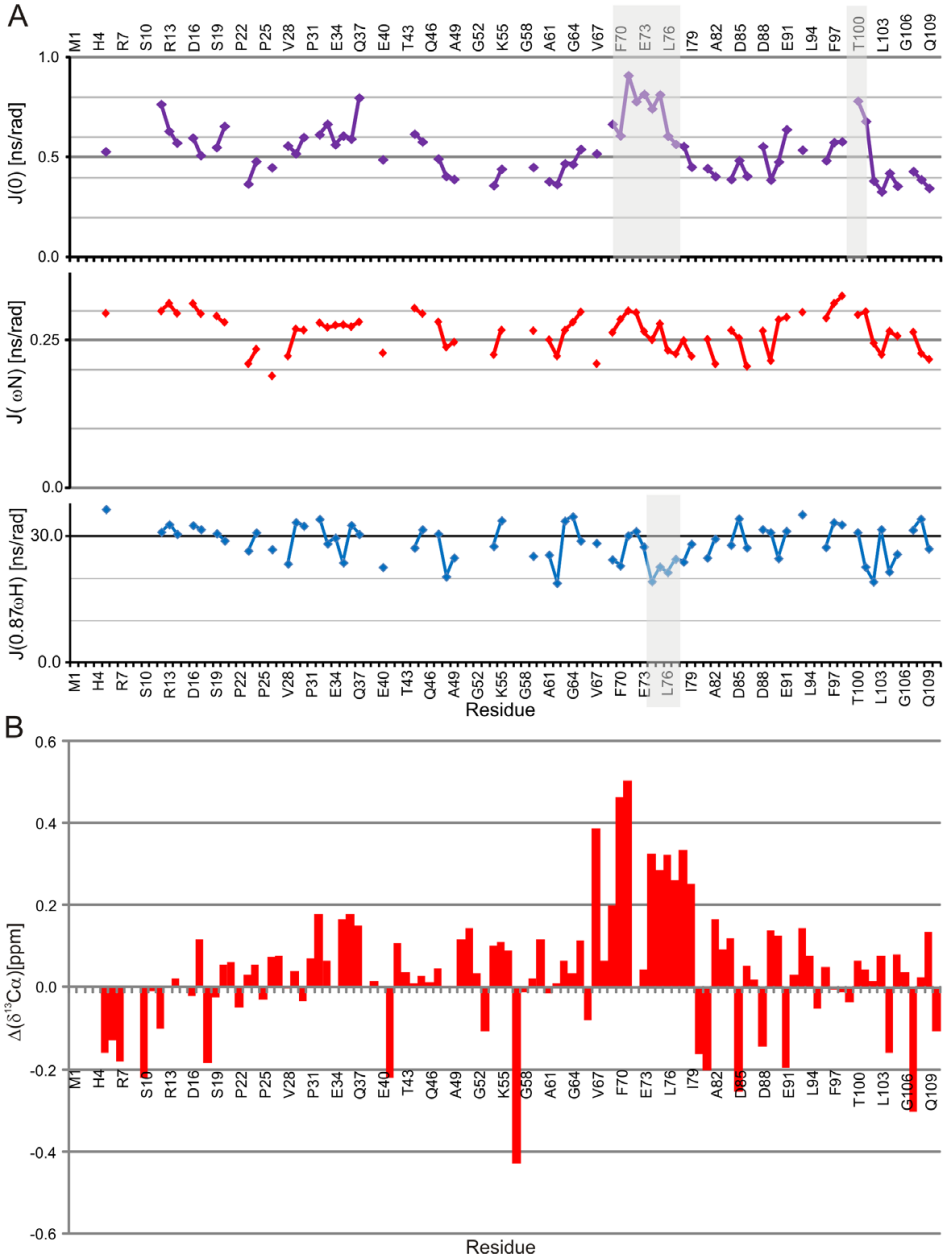
Reduced spectral density plots and effect of pH to transiently populated secondary structures. (**A**) Plots for spectral density at zero frequency, *J*(0), at the ^15^N, *J*(ω_N_), and at the ^1^H, *J*(0.87ω_H_). Regions with suggested transient secondary structure elements are shaded. (**B**) ^13^Cα chemical shift perturbation, at the pH 6.5 subtracted from the shifts at pH 5.

The measured ^15^N *R*_2_ and *R*_1_ rates for PAGE5 are shown in Supplementary Figure S1 and Supplementary Table S1. In particular, experimental ^15^N *R*_2_ rates (average ^15^N *R*_2_∼3.39 s^−1^), measured at 800 MHz ^1^H frequency, are significantly lower than predicted for a globular protein of similar size (^15^N *R*_2_∼11 s^−1^) confirming that PAGE5 is an IDP. Inspection of the *R*_2_/*R*_1_ ratio (Supplementary Figure S1) reveals several residues with elevated *R*_2_/*R*_1_ ratio i.e. their relaxation is dominated by slower time scale motions, implying restricted motional freedom for few segments e.g. ^37^Gln-Glu^38^, ^69^Ala-Phe-Gln-Gln-Glu-Leu-Ala-Leu^76^ and ^100^Thr-Lys^101^ corresponding to the transient structural elements identified using secondary chemical shift and J coupling analysis. A more elaborated relaxation analysis in terms of spectral density mapping at three different frequencies, is shown in Figure 5A. Restricted backbone motion in ps-ns timescales is observed for residues ^69^Ala-Phe-Gln-Gln-Glu-Leu-Ala-Leu-Leu-Lys^78^ as indicated by increased *J*(0) and decreased *J*(0.87ω_H_) spectral densities. Interestingly, however, *J*(0.87ω_H_) values show no significant decrease for ^71^Gln-Gln-Glu^73^ suggesting restricted backbone dynamics or conformational exchange in slower µs timescale. Increased *J*(0) densities can also be seen for residues ^30^Gln-Pro-Thr-Glu-Glu-Lys-Arg-Gln^37^ and ^100^ -Lys^101^. However, the former, highly charged segment, shows no significantly decreased dynamics in the ps timescale as evidenced by relatively uniform *J*(0.87ω_H_) values. 10 out of 12 first N-terminal NH resonances are broadened beyond detection due to increased NH exchange with the solvent. Plausible conformational exchange can be probed for Glu-12 flanking this region. Glu-12 shows an increased *J*(0) value, whereas no concomitant decrease in *J*(0.87ω_H_) is observed, confirming the additional line broadening being caused by µs-ms timescale motion in the N-terminal part of PAGE5. The very C-terminal residues display large amplitude motion in fast ps timescale manifested by very low *J*(0) values as well as large negative heteronuclear NOEs (Figure 2C).

### Hydrodynamic radius indicates PAGE5 exists as a monomer in solution

The molecular weight estimation with SEC remained ambiguous. It was not clear, if PAGE5 exist as monomer, dimer, trimer or tetramer. To further analyze the oligomerization state of PAGE5, we used PG-SLED diffusion NMR experiment for determining the hydrodynamic radius (R_h_) of PAGE5 in solution [43]. By relating apparent translational diffusion rates (D_trans_) measured for PAGE5 and the reference compound 1,4-dioxane, with a known R_h_ = 2.12 Å, according to
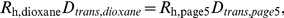
(10)we obtained R_h, page5_∼31.8 Å. This agrees well with a theoretical R_h_ of 30.2 Å for a monomeric IDP, gleaned using a method that takes into account amino acid composition of a protein as described by Marsh and Forman-Kay [44]. It is also comparable to results obtained with other proteins [44].

### PAGE5 DNA binding studies and effect of pH to secondary structure of PAGE5

Although secondary chemical shifts are highly useful for identification of PSEs that is transiently populated helical or extended conformations establishing potential interaction modules, not necessarily all these regions correspond to binding epitopes or MoRFs. DNA binding features of PAGE5 were predicted using the DBS-Pred software package, which predicted probability of PAGE5 to bind DNA is 86%. This, as well as experimental data on homologous PAGE4 protein [12] led us to study plausible DNA binding of PAGE5. To this end, we employed ^15^N-HSQC based approach for monitoring PAGE4-like DNA binding induced chemical shift perturbations on PAGE5 sample upon addition of double stranded DNA fragment pool. In addition we used electrophoretic mobility shift assay (EMSA) for identifying DNA binding, using similar DNA fragments (Supplementary Figure S2). Although we were not able to observe either any perturbations or mobility shift, this does not exclude possibility that PAGE5 recognizes specific DNA sequence.

For studying the effect of pH on structure of PAGE5, we compared the chemical shifts at three different pHs, at 5.0, 6.5 and 8.5, all of which are above the theoretical pI of PAGE5, 4.13. By measuring ^15^N-HSQC spectrum at pH 8.5 where amide proton exchange with solvent is especially pronounced, resulted in disappearance of the vast majority of amide protons and only 23 remained visible, which mainly belong to hydrophobic residues (Figure 2A). However, the chemical shifts of these remaining residues did not change. At acidic conditions (pH 5), amide proton and nitrogen chemical shifts of were significantly altered and also N-terminal HN resonances became visible. Interestingly, comparison of ^13^Cα chemical shifts at pH 5.0 to the corresponding chemical shifts at pH 6.5, underpins increasing propensity for the α-helical conformation at the region of ^65^Thr-Asp-Val-Glu-Ala-Phe-Gln-Gln-Glu-Leu-Ala-Leu-Leu^77^ (Figure 5B). On the contrary, the difference in ^13^Cα chemical shifts between 6.5 and pH 8.5 were insignificant (data not shown). These observations confirm that the α-helical propensity of the segment ^65^Thr-Asp-Val-Glu-Ala-Phe-Gln-Gln-Glu-Leu-Ala-Leu-Leu^77^ further increases at acidic pH. According to Zbilut et al. [45] proteins which fold via transient secondary structures have lower net charge and higher hydrophobicity in comparison to two-state folders [45]. Charge distribution along the primary sequence of PAGE5 is rather uniform, except for the region comprised of residues 19–32, which is free from the charged residues (Supplementary Figure S3). According to hydropathy score plot, hydrophobicity of the PAGE5 is highest at the regions encompassing residues 22–27 and 71–81. The lowering pH decreases the net charge of the latter region (71–81) and may explain the increased α-helical propensity observed by the chemical shift analysis. If transient secondary structured regions serve as MoRFs, decreased intracellular pH of the cancer cell may have biologically significance, promoting interactions between natively disordered PAGE5 and its binding partner.

### Conclusion

Taken together, in the present study we have shown using the experimental data at single residue resolution level that PAGE5, a member of GAGE family proteins, is an intrinsically highly disordered protein. However, there are few regions with predominant secondary structure propensities, i.e. ^65^Thr-Asp-Val-Glu-Ala-Phe-Gln-Gln-Glu-Leu-Ala-Leu-Leu^77^ as well as ^97^Phe-Glu-Pro-Thr-Lys-Val^102^ showing propensity to form α-helical conformations. These regions were identified using secondary chemical shift, J coupling, relaxation as well as H/D exchange data concomitantly. Although propensities for these secondary structures elements are low, the segment ^65^Thr-Asp-Val-Glu-Ala-Phe-Gln-Gln-Glu-Leu-Ala-Leu-Leu^77^ was predicted by PSIPRED algorithm. Less compelling evidence of transient extended conformation can be found for residues ^29^Gln-Gln-Pro-Thr-Glu-Glu-Lys-Arg-Gln-Glu-Glu-Glu-Pro-Pro^42^, if present, the population is low. It is plausible that these transiently populated secondary structure regions serve as PSEs or MoRFs for PAGE5 thus being potential interaction sites for the natural binding partners of PAGE5 in cancer cells and in germ line cells. Interestingly, we also found that at acidic pH, the MoRF region (^65^Thr-Asp-Val-Glu-Ala-Phe-Gln-Gln-Glu-Leu-Ala-Leu-Leu^77^) more prominently populates α-helical secondary structure as compared to neutral pH (6.5). This study also illustrates how solution state NMR spectroscopy can be utilized for characterization of unfolded proteins and recognition of transiently populated conformations at single residue resolution.

## Materials and Methods

### NMR sample preparation

Gene encoding variant 2 of CT16 (GeneBank accession code NM_001013435) was cloned into a pGEX-2T as described previously [17]. ^13^C, ^15^N labelled PAGE5 was expressed in *Eschericia coli* BL21, using 2 g/l ^13^C D-glucose and 1 g/l ^15^NH_4_Cl, as sole carbon and nitrogen sources, respectively. Glutathione-S-transferase (GST) fused PAGE5 was purified and thrombin cleaved as described earlier [17]. Cleaved PAGE5 was applied into the Superdex S75 size-exclusion column with NMR buffer, containing 20 mM sodium phosphate, 50 mM NaCl, pH 6.5. Fractions containing PAGE5 protein were pooled and concentrated by using Vivaspin2 centrifugal concentrator (MWCO = 2 kDa) to final protein concentration of 1 mM. Prior to NMR measurements 7% of D_2_O was supplemented into the sample. Protein concentrations were measured using Bio-Rad Protein Assay (Bio-Rad) based on the method of Bradford, using bovine serum albumin (BSA) as a reference. NMR samples with 7% D_2_0, were also prepared at varied pHs, i.e. 20 mM Bis-Tris, pH 5 and 20 mM Tris-HCl, pH 8.5.

### Preparation of dsDNA pool and EMSA experiment

The degenerate dsDNA pool was prepared by PCR using the primer and template sequences described in [12]. The template contained a 10-base degenerate stretch of any of the four nucleotides. In addition, a corresponding template having a 10-base stretch of nucleotides G or C was designed and used to prepare a GC-rich dsDNA pool. To label the dsDNA pools for EMSA, the PCR amplification was repeated in the presence of 67 nM [α-^32^P]dCTP. EMSA was performed by incubating 20-µl reactions containing 10 µM [α-^32^P]dCTP-labeled degenerate dsDNA pool; 80, 40, 20 or 0 µM PAGE5; 10% (v/v) glycerol; 50 mM KCl; and 20 mM HEPES (pH 7.4) at RT for 40 min and running them on a 6% polyacrylamide gel in TBE buffer (pH 8.3). The dried gel was visualized using a Fuji BAS-1800 phosphorimager.

### Bio-informatics

The DBS-Pred (http://gibk26.bio.kyutech.ac.jp/jouhou/shandar/netasa/dbs-pred/) [46] was used to analyze DNA binding site of PAGE5, where the level of sensitivity was selected as Medium. The software IUPred (http://iupred.enzim.hu/) was used for prediction of disordered parts of PAGE proteins [47] and ANCHOR (http://anchor.enzim.hu/) for prediction of MoRFs [48]. PSIPRED server (http:// http://bioinf.cs.ucl.ac.uk/psipred/) was employed to predict secondary structure elements in PAGE5.

### NMR data collection and processing

All spectra were acquired at 25°C using a Varian Unity INOVA 800 MHz spectrometer equipped with a 5 mm {^15^N,^13^C}^1^H triple-resonance x,y,z-gradient probehead or 5 mm {^15^N,^13^C}^1^H triple-resonance z-gradient coldprobe, and a Varian Unity INOVA 600 MHz spectrometer, equipped either with a 5 mm {^15^N,^13^C}^1^H triple-resonance z-gradient coldprobe or {^15^N,^13^C}^1^H triple-resonance z-gradient probe. The double- and triple-resonance experiments performed for the sequence-specific backbone and partial side-chain assignments included 2D ^15^N-HSQC, ^13^C-HSQC, ^13^C-(CT)-HSQC, 3D CBCA(CO)NH [27], [49], iHNCACB [26], i(HCA)CO(CA)NH [29], HNCO [30], Hα detected HCAN, HCA(CO)N [50], i(HCA)CON, (HCA)CON(CA)H and (HCA)NCO(CA)H [31], [32]. Spin-lattice relaxation rates (^15^N *R*_1_), spin-spin relaxation rates (^15^N *R*_2_) and steady-state heteronuclear {^1^H}-^15^N NOEs were determined using the methods described in [51]. For ^15^N *R*_1_, ten 2D ^15^N-HSQC spectra [51], with relaxation delays of 10, 50, 90, 150, 250, 400, 650, 1000, 1300 and 1600 ms were acquired and for ^15^N *R*_2_, nine ^15^N-HSQC spectra by using relaxation delays of 10, 50, 110, 150, 190, 250, 330, 390 and 450 ms. For measuring heteronuclear {^1^H}-^15^N NOE values, NOE mixing time of 3 s was used. ^1^J and ^2^J couplings between ^15^N(*i*) and ^13^Cα(*i*) and ^15^N(*i*) and ^13^Cα*i*-1) spins were measured using the 3D HNCO E.COSY type experiment [36]. The CLEANEX experiment was measured using the pulse sequence described in [38]. Translational diffusion rates (D_trans_) were measured PG-SLED sequence [43], using 1,4-dioxane as a reference molecule, dissolved in one solution. Thirty 1D 1H PG-SLED spectra were acquired, with gradient strengths ranging from 1.8 G/cm to 56.8 G/cm. The integrated peak volumes were fitted to a single Gaussian to yield D_trans_ values for Page5 and 1,4-dioxane.

DNA titrations were performed with constant 40 µM dsDNA concentration, with increasing ^13^C, ^15^N labelled PAGE5 concentration from 10 µM to 40 µM. Buffer used for titration experiment was 20 mM Bis-Tris, pH 6.5.

Spectra were processed using VNMR 6.1C and VNMRJ 2.1C software packages (Varian Inc., Palo Alto, CA) and analyzed by Sparky [52].

## Supporting Information

Table S1 ^15^N R_1_ and R_2_ relaxation rates, {^1^H}-^15^N heteronuclear NOE, heteronuclear ^1^JNC and ^2^JNC couplings, and chemical shifts of PAGE5.
(PDF)

Figure S1 ^15^N R_1_ and R_2_ relaxation rates and ratio of R_2_/R_1_ of PAGE5 plotted as a function of primary structure.
(PDF)

Figure S2 DNA binding test by EMSA. Lanes 1 to 3, 10 µM dsDNA pool containing 10-bp stretch of S nucleotides (S probe) incubated with 80, 40 and 20 µM CT16. Lanes 4 to 6, 10 µM dsDNA pool containing 10-bp stretch of N nucleotides (N probe) incubated with 80, 40 and 20 µM CT16. Lanes 10 to 12; 1, 0.1 and 0.01 µM S probe. Lanes 13 to 15; 1, 0.1 and 0.01 µM N probe. The lanes 7 to 9 are empty. Equal volumes were loaded.
(PDF)

Figure S3 Hydropathy Score and charge distribution at pH 5 and 6.5 plotted as a function of primary structure. The most hydrophobic regions are shaded.
(PDF)

## References

[1] Simpson AJ, Caballero OL, Jungbluth A, Chen YT, Old LJ (2005). Cancer/testis antigens, gametogenesis and cancer.. Nat Rev Cancer.

[2] Fratta E, Corala S, Covrea A, Parisia G, Colizzia F (2011). The biology of cancer testis antigens: Putative function, regulation and therapeutic potential.. Molecularoncology.

[3] Ross MT, Grafham DV, Coffey AJ, Scherer S, McLay K (2005). The DNA sequence of the human X chromosome.. Nature.

[4] Hofmann O, Caballero OL, Stevenson BJ, Chen YT, Cohen T (2008). Genome-wide analysis of cancer/testis gene expression.. Proc Natl Acad Sci U S A.

[5] Davis ID, Chen W, Jackson H, Parente P, Shackleton M (2004). Recombinant NY-ESO-1 protein with ISCOMATRIX adjuvant induces broad integrated antibody and CD4(+) and CD8(+) T cell responses in humans.. Proc Natl Acad Sci U S A.

[6] Brichard VG, Lejeune D (2007). GSK's antigen-specific cancer immunotherapy programme: pilot results leading to Phase III clinical development.. Vaccine.

[7] Odunsi K, Qian F, Matsuzaki J, Mhawech-Fauceglia P, Andrews C (2007). Vaccination with an NY-ESO-1 peptide of HLA class I/II specificities induces integrated humoral and T cell responses in ovarian cancer.. Proc Natl Acad Sci U S A.

[8] Slingluff CL, Petroni GR, Chianese-Bullock KA, Smolkin ME, Hibbitts S (2007). Immunologic and clinical outcomes of a randomized phase II trial of two multipeptide vaccines for melanoma in the adjuvant setting.. Clin Cancer Res.

[9] Vansteenkiste JF, Zielinski M, Dahabreh IJ, Linder A, Lehmann F (2008). Association of gene expression signature and clinical efficacy of MAGE-A3 antigenspecific cancer immunotherapeutic (ASCI) as adjuvant therapy in resected stage IB/II non-small cell lung cancer (NSCLC).. J Clin Oncol.

[10] Almeida LG, Sakabe NJ, deOliveira AR, Silva MC, Mundstein AS (2009). CTdatabase: a knowledge-base of high-throughput and curated data on cancer-testis antigens.. Nucleic Acids Research,.

[11] Zendman AJ, Van Kraats AA, Weidle UH, Ruiter DJ, van Muijen GN (2002). The XAGE family of cancer/testisassociated genes: alignment and expression profile in normal tissues, melanoma lesions and Ewing's sarcoma.. Int J Cancer.

[12] Zeng Y, He Y, Yang F, Mooney SM, Getzenberg RH (2011). The Cancer/Testis Antigen Prostate-associated Gene 4 (PAGE4) is a highly intrinsically disordered protein.. J Biol Chem.

[13] Cilensek ZM, Yehiely F, Rupinder K, Kular RK, Deiss LP (2002). A Member of the GAGE Family of Tumor Antigens is an Anti-Apoptotic Gene That Confers Resistance to Fas/CD95/APO-1, Interferon-γ, Taxol and γ-Irradiation.. Cancer Biology & Therapy.

[14] Chen ME, Lin SH, Chung LW, Sikes RA (1998). Isolation and characterization of PAGE-1 and GAGE-7. New genes expressed in the LNCaP prostate cancer progression model that share homology with melanoma associated antigens.. J Biol Chem.

[15] Brinkmann U, Vasmatzis G, Lee B, Yerushalmi N, Essand M (1998). PAGE-1, an X chromosome-linked GAGE-like gene that is expressed in normal and neoplastic prostate, testis, and uterus.. Proc Natl Acad Sci U S A.

[16] Scanlan MJ, Gordon CM, Williamson B, Lee SY, Chen YT (2002). Identification of cancer/testis genes by database mining and mRNA expression analysis.. Int J Cancer.

[17] Rappu P, Nylund C, Ristiniemi N, Kulpakko J, Vihinen P (2011). Detection of melanoma-derived cancer-testis antigen CT16 in patient sera by novel immunoassay.. Int J Cancer.

[18] Uversky VN (2010). The mysterious unfoldome: Structureless, underappreciated, yet vital part of any given proteome.. J Biomed Biotech.

[19] Hazy E, Tompa P (2009). Limitations of Induced Folding in Molecular Recognition by Intrinsically Disordered Proteins.. Chem Phys Chem.

[20] Ma B, Nussinov R (2010). Enzyme dynamics point to stepwise conformational selection in catalysis.. Curr Opin Chem Biol.

[21] Tompa P, Fuxreiter M (2007). Fuzzy complexes: polymorphism and structural disorder in protein–protein interactions.. TIBS.

[22] Fuxreiter M, Simon I, Friedrich P, Tompa P (2004). Preformed structural elements feature in partner recognition by intrinsically unstructured proteins.. J Mol Biol.

[23] Vasic V, Oldfield CJ, Mohan A, Radivojac P, Cortese MS (2007). Characterization of molecular recognition features, MoRFs, and their binding partners.. J Proteome Res.

[24] Neduva V, Russell RB (2005). Linear motifs: evolutionary interaction switches.. FEBS Lett.

[25] Tompa P, Szasz C, Buday L (2005). Structural disorder throws new light on moonlighting.. Trends Biochem Sci.

[26] Tossavainen H, Permi P (2004). Optimized pathway selection in intraresidual triple-resonance experiments.. J Magn Reson.

[27] Grzesiek S, Ikura M, Clore GM, Gronenborn AM, Bax A (1992). An efficient experiment for sequential backbone assignment of medium-sized isotopically enriched proteins.. J Magn Reson.

[28] Yao J, Dyson JH, Wright PE (1997). Chemical shift dispersion and secondary structure prediction in unfolded and partly folded proteins.. FEBS Lett.

[29] Mäntylahti S, Tossavainen H, Hellman M, Permi P (2009). An intraresidual i(HCA)CO(CA)NH experiment for the assignment of main-chain resonances in 15N, 13C labeled proteins.. J Biomol NMR.

[30] Muhandiram DR, Kay LE (1994). Gradient-enhanced triple-resonance three-dimensional NMR experiments with improved sensitivity.. J Magn Reson.

[31] Mäntylahti S, Aitio O, Hellman M, Permi P (2010). HA-detected experiments for the backbone assignment of intrinsically disordered proteins.. J Biomol NMR.

[32] Mäntylahti S, Hellman M, Permi P (2011). Extension of the HA-detection based approach: (HCA)CON(CA)H and (HCA)NCO(CA)H experiments for the main-chain assignment of intrinsically disordered proteins.. J Biomol NMR.

[33] Wishart DS, Bigam CG, Yao J, Abilgaard F, Dyson HJ (1995). 1H, 13C and 15N chemical shift referencing in biomolecular NMR.. J Biomol NMR.

[34] Kjaergaard M, Poulsen F (2011). Sequence correction of random coil chemical shifts: correlation between neighbor correction factors and changes in the Ramachandran distribution.. J Biomol NMR.

[35] Marsh JA, Singh VK, Jia Z, Forman-Kay JD (2006). Sensitivity of secondary structure propensities to sequence differences between α- and γ-synuclein: Implications for fibrillation.. Protein Sci.

[36] Puttonen E, Tossavainen H, Permi P (2006). Simultaneous determination of one- and two-bond scalar and residual dipolar couplings between 13C′, 13Calpha and 15N spins in proteins.. Magn Reson Chem.

[37] Wirmer J, Schwalbe H (2002). Angular dependence of 1J(Ni,Calphai) and 2J(Ni,Calpha(i-1)) coupling constants measured in J-modulated HSQCs.. J Biomol NMR.

[38] Hwang TL, van Zijl PC, Mori S (1998). Accurate quantitation of water–amide proton exchange rates using the phase-modulated CLEAN chemical EXchange (CLEANEX-PM) approach with a Fast-HSQC (FHSQC) detection scheme.. J Biomol NMR.

[39] Farrow NA, Zhang O, Szabo A, Torchia DA, Kay LE (1995). Spectral density function mapping using 15N relaxation data exclusively.. J Biomol NMR.

[40] Lipari G, Szabo A (1982). Model-free approach to the interpretation of nuclear magnetic resonance relaxation in macromolecules. Theory and range of validity.. J Am Chem Soc.

[41] Lefèvre J-F, Dayie KT, Peng JW, Wagner G (1996). Internal mobility in the partially folded DNA binding and dimerization domains of GAL4: NMR analysis of the N-H spectral density functions.. Biochemistry.

[42] Atkinson RA, Lefèver J-F (1999). Reduced spectral density mapping for proteins: Validity for studies of 13C relaxation.. J Biomol NMR.

[43] Jones JA, Wilkins DA, Smith LJ, Dobson CM (1997). Characterization of protein unfolding by NMR diffusion measurements.. J Biomol NMR.

[44] Marsh JA, Forman-Kay JD (2010). Sequence determinants of compaction in intrinsically disordered proteins.. Biophys J.

[45] Zbilut JP, Giuliani A, ColosimoA, Mitchell JC, Colafranceschi M, Marwan N, Webber CL, Uversky VN (2004). Charge and hydrophobicity patterning along the sequence predicts the folding mechanism and aggregation of proteins: A computational approach.. J Proteome Res.

[46] Ahmad S, Gromiha MM, Sarai A (2004). Analysis and prediction of DNA-binding proteins and their binding residues based on composition, sequence and structural information.. Bioinformatics.

[47] Dosztányi Z, Csizmók V, Tompa P, Simon I (2005). IUPred: web server for the prediction of intrinsically unstructured regions of proteins based on estimated energy content.. Bioinformatics.

[48] Dosztányi Z, Mészáros B, Simon I (2009). ANCHOR: web server for predicting protein binding regions in disordered proteins.. Bioinformatics.

[49] Sattler M, Schleucher J, Griesinger C (1999). Heteronuclear multidimensional NMR experiments for the structure determination of proteins in solution employing pulsed field gradients.. Prog Nucl Magn Reson Spectr.

[50] Wang AC, Grzesiek S, Tschudin R, Lodi PJ, Bax A (1995). Sequential backbone assignment of isotopically enriched proteins in D_2_O by deuterium-decoupled HA(CA)N and HA(CACO)N.. J Biomol NMR.

[51] Farrow NA, Muhandiram R, Singer AU, Pascal SM, Kay CM (1994). Backbone dynamics of a free and phosphopeptide-complexed Src homology 2 domain studied by 15N NMR relaxation.. Biochemistry.

[52] Goddard TD, Kneller DG (2002). Sparky 3.

