# Supplementary material for: Characterization of Intrinsically Disordered Prostate Associated Gene (PAGE5) at Single Residue Resolution by NMR Spectroscopy
Source: PLoS One. 2011 Nov 2;6(11):e26633. doi: 10.1371/journal.pone.0026633 (PMC3206799; doi:10.1371/journal.pone.0026633)
Supplement: Table S1 — 15N R1 and R2 relaxation rates, {1H}-15N heteronuclear NOE, heteronuclear 1JNC and 2JNC couplings, and chemical shifts of PAGE5. (PDF) [file pone.0026633.s001.pdf]

Residue	R1 [1/s]	R2 [1/s]	HetNOE	1JNCα [Hz]	2JNCα [Hz]
M1					
S2					
E3					
H4					
V5	2.19	3.46	-0.07		
T6	2.25	3.88			
R7					
S8					
Q9					
S10					
S11					
E12	2.17	4.45	0.08		
R13	2.27	3.93	0.07		
G14	2.14	3.60	0.08		
N15	2.34	4.05			7.77
D16	2.26	3.78	0.07	10.52	7.65
Q17	2.14	3.34	0.05	10.47	
E18					7.96
S19	2.11	3.50	0.06	11.46	
S20	2.03	3.90	0.08		7.47
Q21	2.08	3.80		11.35	
P22					8.87
V23	1.56	2.43	-0.10	10.72	7.98
G24	1.75	3.02	-0.13	12.71	
P25					8.85
V26	1.43	2.72	-0.21	10.68	7.90
I27				10.47	7.99
V28	1.62	3.26	0.07	10.55	8.36
Q29	1.99	3.31	-0.08	10.99	8.53
Q30	1.97	3.65	-0.06	11.17	
P31					9.09
T32	2.06	3.76	-0.06	11.48	7.59
E33	1.97	3.92	0.08	10.73	
E34	2.00	3.50	0.05		8.06
K35	1.96	3.65	0.22	10.86	8.32
R36	2.01	3.64	-0.05	10.88	8.38
Q37	2.04	4.52	0.04	10.85	8.12
E38	1.87	4.21		10.70	
E39					8.12
E40	1.64	2.98	0.11	11.22	
P41					
P42					9.14
T43	1.92	3.34		11.48	7.54
D44	2.17	3.80	0.19	10.83	8.17
N45	2.14	3.64	0.05	10.29	7.59
Q46	2.17	3.58		10.64	7.70
G47	2.05	3.22	0.04	11.40	8.32
I48	1.69	2.64	0.22	10.81	7.62
A49	1.78	2.63	0.10	11.22	

P50					8.58
S51	2.10	3.53		11.38	
G52	2.15	3.23			8.35
E53	2.10	3.91		11.07	8.06
I54	1.67	2.45	-0.07	10.54	7.83
K55	1.98	2.98	-0.10	10.80	
N56	2.21	3.55			8.74
E57	2.11	3.88		10.13	7.86
G58	2.05	3.35		11.72	8.36
A59	1.91	2.96	0.15	11.54	
P60					8.95
A61	1.81	2.61	0.09	10.96	8.37
V62	1.58	2.41	0.23	10.80	7.73
Q63	1.98	3.10	-0.09	10.84	9.00
G64	2.08	3.13	-0.08	11.43	8.93
T65	2.14	3.47	0.13	11.59	7.29
D66	2.18	3.32		10.55	7.70
V67	1.57	3.09	-0.16	10.51	7.18
E68				10.52	7.51
A69	1.88	3.86	0.16	10.46	7.63
F70	2.01	3.68	0.26	10.78	7.60
Q71	2.16	5.06	0.10	10.47	7.78
Q72	2.15	4.50	0.07	10.57	
E73	1.92	4.53	0.08		7.52
L74	1.76	4.11	0.29	10.28	7.38
A75	1.96	4.53	0.25	10.39	7.67
L76	1.66	3.49	0.17	10.33	7.44
L77	1.65	3.32	0.04	10.62	7.88
K78	1.79	3.34	0.14	10.72	8.25
I79	1.65	2.84	-0.10	10.38	7.72
E80	1.75	3.30		10.73	8.17
D81	1.81	2.88	0.11	10.88	7.91
A82	1.58	2.61	-0.20	11.15	
P83					8.74
G84	1.93	2.72	0.07	11.82	8.67
D85	1.90	3.12	-0.16	11.01	8.04
G86	1.54	2.59	-0.14	12.87	
P87					8.25
D88	1.96	3.44	-0.04	10.68	7.81
V89	1.63	2.56	-0.22	10.38	
R90	2.02	3.13	0.21		
E91	2.10	3.88	0.04		8.51
G92	2.14	3.59		11.28	8.53
T93	2.19	3.49	-0.03	11.56	7.44
L94				11.26	
P95					9.03
T96	2.06	3.18	0.14	11.53	7.52
F97	2.27	3.69	0.06	11.17	8.11
D98	2.35	3.74	0.10	10.13	
P99					6.92

T100	2.13	4.50	0.06		10.47	6.32
K101	2.09	4.02	0.30		10.60	7.60
V102	1.72	2.56	0.28		10.51	7.65
L103	1.70	2.35	-0.20		10.47	7.89
E104	1.87	2.80	0.26		10.63	7.91
A105	1.85	2.53	0.10		10.75	8.25
G106	1.85	2.71			11.79	8.61
E107	1.94	2.91	-0.04		10.80	8.07
G108	1.73	2.63	-0.27		11.51	8.33
Q109	1.61	2.37	-0.08		10.86	7.72
L110	0.77		-0.39		10.08	

Residue	δC' [ppm]	δCα [ppm]	δHα [ppm]	δN [ppm]	δCβ [ppm]	δHN [ppm]
M1	176.50		4.51	122.00		
S2	174.60		4.38	116.20		
E3	176.30		4.21	122.30		
H4	175.35	56.40	4.59	119.70	30.60	
V5	176.31	62.60	4.09	121.20	32.90	8.04
T6	174.58	62.30	4.26	118.00	69.80	8.32
R7	176.34	56.30	4.35	123.50	30.90	8.42
S8	174.70		4.39	116.80		
Q9	176.20		4.36	122.00		
S10	174.80		4.43	116.30		
S11	174.66	58.70	4.43	117.40	63.80	
E12	176.54	56.90	4.25	122.30	30.20	8.45
R13	176.86	56.30	4.31	121.00	30.90	8.36
G14	173.94	45.40	3.96	109.30	-	8.43
N15	175.25	53.30	4.71	118.40	39.00	8.42
D16	176.42	54.70	4.54	120.30	41.00	8.47
Q17	176.32	56.20	4.27	119.40	29.40	8.35
E18	176.79	57.00	4.24	121.30	30.20	8.45
S19	174.65	58.50	4.45	116.10	63.90	8.37
S20	174.18	58.40	4.44	117.40	63.90	8.35
Q21	173.89	53.70	4.6	122.40	29.00	8.32
P22	176.88	63.20	4.42	-	32.10	-
V23	176.43	62.40	4.09	120.10	32.90	8.34
G24	171.54	44.50	4.14	112.30	-	8.3
P25	176.84	63.00	4.4	133.90	32.20	-
V26	176.14	62.60	4.01	120.60	32.70	8.28
I27	176.06	60.70	4.12	126.00	38.60	8.35
V28	175.84	62.20	4.08	125.60	32.80	8.35
Q29	175.55	55.60	4.3	124.80	29.60	8.53
Q30	174.10	53.80	4.57	123.30	28.80	8.57
P31	177.09	63.30	4.46	137.00	32.20	-
T32	174.57	62.00	4.26	114.40	70.00	8.3
E33	176.27	56.50	4.27	122.80	30.60	8.47
E34	176.28	56.60	-	122.50	30.40	8.48
K35	176.40	56.10	4.28	123.10	33.00	8.42
R36	176.33	56.10	4.29	123.30	30.90	8.52
Q37	175.94	56.10	4.27	122.20	29.40	8.6
E38	176.31	56.70	4.25	122.00	30.40	8.58
E39	176.20	56.30	4.25	121.30	30.60	8.44
E40	174.08	54.40	4.53	123.30	29.80	8.47
P41	174.70		-	-	-	-
P42	177.29	63.00	4.48	135.00	32.20	-
T43	174.44	61.90	4.27	113.90	70.10	8.33
D44	176.07	54.20	4.59	122.10	41.30	8.4
N45	175.49	53.50	4.65	119.00	38.80	8.47
Q46	176.55	56.40	4.24	119.70	29.20	8.48
G47	173.91	45.40	3.91	109.00	-	8.44
I48	175.82	60.80	4.13	119.60	38.90	7.94
A49	175.53	50.50	4.57	129.60	18.20	8.46

P50	177.14	63.20	4.42	135.30	32.10 -	
S51	175.25	58.50	4.39	115.70	64.00	8.46
G52	173.94	45.30	3.94	110.40 -		8.45
E53	176.34	56.50	4.24	120.30	30.50	8.26
I54	176.17	61.10	4.09	122.90	38.50	8.32
K55	175.97	56.00	4.31	126.10	33.20	8.52
N56	175.14	53.20	4.68	120.60	39.10	8.59
E57	176.81	56.90	4.25	121.80	30.30	8.58
G58	173.41	45.10	3.9	109.50 -		8.45
A59	175.49	50.50	4.56	124.60	18.20	8.13
P60	176.63	63.00	4.36	135.30	32.10 -	
A61	177.82	52.40	4.27	124.20	19.20	8.43
V62	176.17	62.20	4.05	119.40	32.90	8.18
Q63	176.33	55.90	4.33	124.30	29.70	8.56
G64	174.29	45.40	4.01	110.40 -		8.55
T65	174.44	62.00	4.31	112.70	70.00	8.15
D66	176.41	54.50	4.6	122.50	41.00	8.51
V67	176.61	62.90	4.01	119.50	32.70	8.05
E68	176.74	57.20	4.15	123.30	30.00	8.42
A69	177.96	53.10	4.19	123.90	19.10	8.2
F70	176.06	58.20	4.53	118.80	39.30	8.14
Q71	176.22	56.30	4.16	121.20	29.20	8.21
Q72	176.41	56.70	4.16	121.00	29.40	8.32
E73	177.10	57.30	4.16	121.40	29.90	8.47
L74	177.67	55.80	4.19	122.20	42.20	8.15
A75	177.91	53.00	4.21	123.30	18.90	8.15
L76	177.35	55.30	4.25	119.90	42.30	7.96
L77	176.97	55.20	4.27	122.00	42.50	7.99
K78	176.19	56.00	4.21	122.50	33.00	8.21
I79	176.34	61.20	4.1	123.30	38.60	8.27
E80	175.97	56.50	4.26	124.80	30.50	8.59
D81	175.49	54.40	4.53	121.60	41.30	8.36
A82	175.57	50.50	4.57	125.00	18.30	8.27
P83	177.73	63.50	4.39	135.40	32.20 -	
G84	173.85	45.20	3.98	108.70 -		8.45
D85	176.48	54.30	4.65	119.90	41.60	8.29
G86	171.99	44.80	4.12	108.60 -		8.27
P87	176.76	63.40	4.37	133.30	32.20 -	
D88	176.52	54.40	4.55	119.70	41.00	8.51
V89	176.31	62.50	4.07	120.30	32.50	8.04
R90	176.48	56.20	4.3	123.90	30.90	8.39
E91	177.00	57.00	4.22	121.80	30.30	8.48
G92	174.23	45.40	3.95	109.70 -		8.49
T93	174.44	61.90	4.28	113.50	70.10	8.05
L94	175.35	53.20	4.61	125.90	41.90	8.36
P95	176.88	63.20	4.43	135.90	32.10 -	
T96	174.11	61.90	4.22	113.80	70.00	8.18
F97	174.32	57.60	4.57	122.70	40.00	8.25
D98	174.82	50.90 -		124.40	41.70	8.26
P99	177.58	63.90	4.3	136.20	32.30 -	

T100	175.12	63.10	4.19	111.20	69.50	8.32
K101	176.40	56.10	4.3	122.40	32.90	7.68
V102	176.11	62.70	4.01	121.10	32.60	7.93
L103	177.39	55.30	4.31	125.50	42.40	8.31
E104	176.25	56.50	4.22	121.80	30.30	8.41
A105	178.27	52.80	4.27	124.70	19.30	8.38
G106	174.30	45.30	3.93	107.80	-	8.38
E107	177.22	56.90	4.26	120.20	30.30	8.36
G108	173.91	45.50	3.9	109.70	-	8.56
Q109	175.04	55.70	4.32	119.60	29.70	8.19
L110		56.90	4.16	129.30	43.42	8
