# Supplementary material for: Characterization of Intrinsically Disordered Prostate Associated Gene (PAGE5) at Single Residue Resolution by NMR Spectroscopy
Source: PLoS One. 2011 Nov 2;6(11):e26633. doi: 10.1371/journal.pone.0026633 (PMC3206799; doi:10.1371/journal.pone.0026633)

Supplementary Figure 1

^{15}N R1 and R2 relaxation rates and ratio of R2/R1of PAGE5 plotted as a function of primary structure.

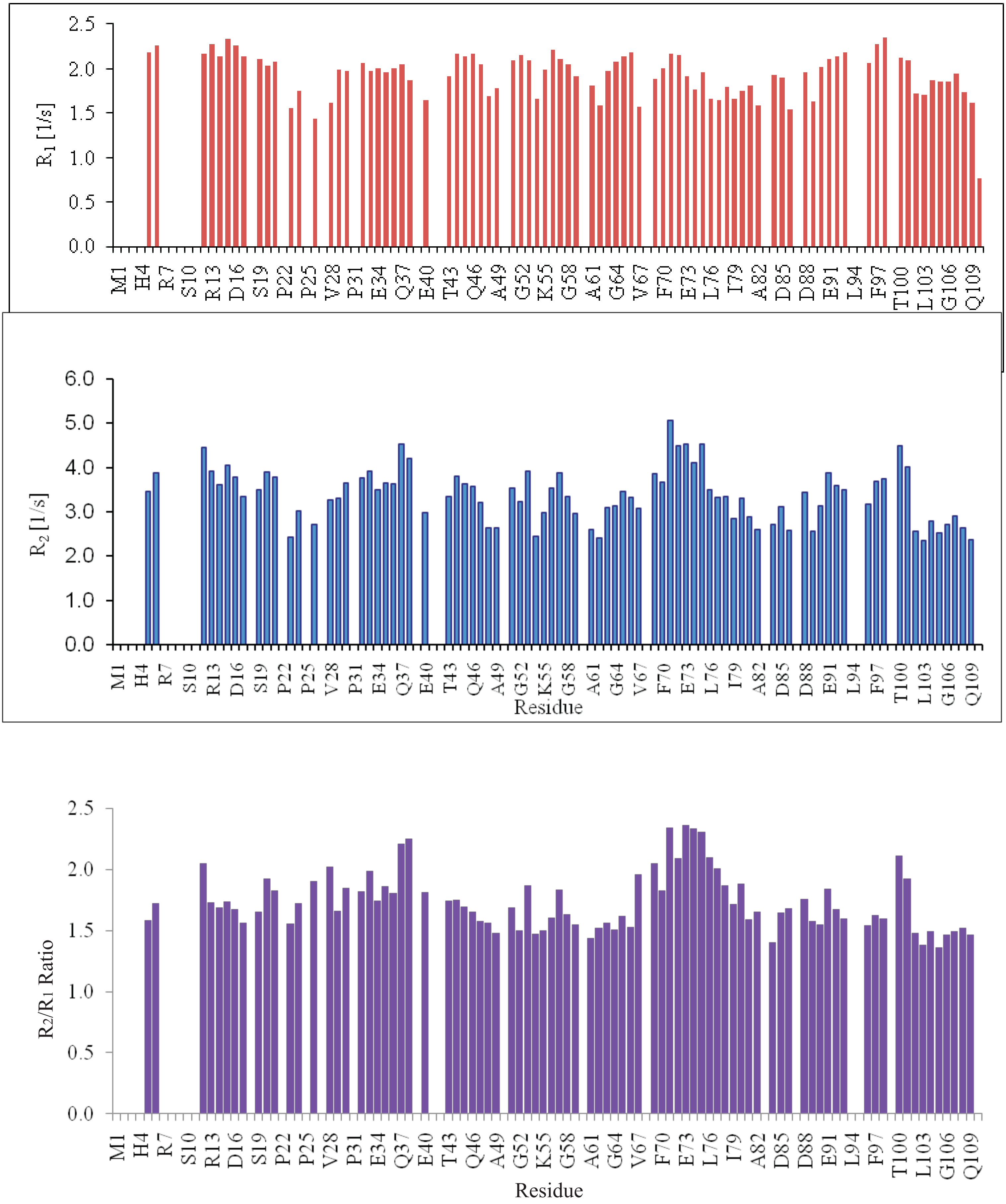

Supplement: Figure S1 — 15N R1 and R2 relaxation rates and ratio of R2/R1 of PAGE5 plotted as a function of primary structure. (PDF) [file pone.0026633.s002.pdf]
