# Supplementary material for: Characterization of Intrinsically Disordered Prostate Associated Gene (PAGE5) at Single Residue Resolution by NMR Spectroscopy
Source: PLoS One. 2011 Nov 2;6(11):e26633. doi: 10.1371/journal.pone.0026633 (PMC3206799; doi:10.1371/journal.pone.0026633)
Supplement: Figure S2 — DNA binding test by EMSA. Lanes 1 to 3, 10 µM dsDNA pool containing 10-bp stretch of S nucleotides (S probe) incubated with 80, 40 and 20 µM CT16. Lanes 4 to 6, 10 µM dsDNA pool containing 10-bp stretch of N nucleotides (N probe) incubated with 80, 40 and 20 µM CT16. Lanes 10 to 12; 1, 0.1 and 0.01 µM S probe. Lanes 13 to 15; 1, 0.1 and 0.01 µM N probe. The lanes 7 to 9 are empty. Equal volumes were loaded. (PDF) [file pone.0026633.s003.pdf]

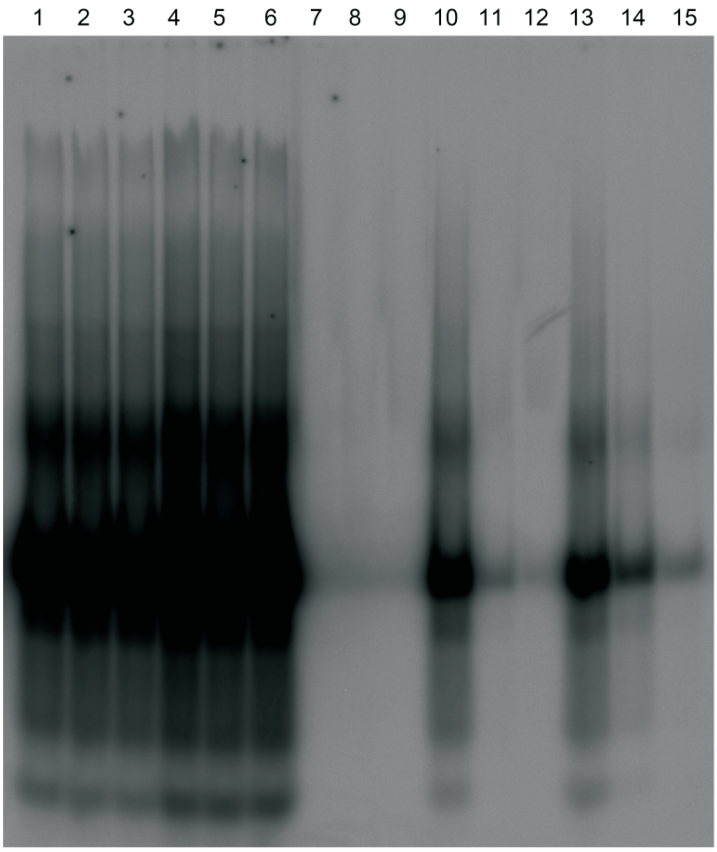

DNA binding test by EMSA. Lanes 1 to 3, 10 μM dsDNA pool containing 10-bp stretch of S nucleotides (S probe) incubated with 80, 40 and 20 μM CT16. Lanes 4 to 6, 10 μM ds DNA pool containing 10-bp stretch of N nucleotides (N probe) incubated with 80, 40 and 20 μM CT16. Lanes 10 to 12; 1, 0.1 and 0.01 μM S probe. Lanes 13 to 15; 1, 0.1 and 0.01 μM N probe. The lanes 7 to 9 are empty. Equal volumes were loaded.
