# Supplementary material for: Characterization of Intrinsically Disordered Prostate Associated Gene (PAGE5) at Single Residue Resolution by NMR Spectroscopy
Source: PLoS One. 2011 Nov 2;6(11):e26633. doi: 10.1371/journal.pone.0026633 (PMC3206799; doi:10.1371/journal.pone.0026633)
Supplement: Figure S3 — Hydropathy Score and charge distribution at pH 5 and 6.5 plotted as a function of primary structure. The most hydrophobic regions are shaded. (PDF) [file pone.0026633.s004.pdf]

Supplementary Figure 3
Hydropathy Score and charge distribution at pH 5 and 6.5 plotted as a function of primary structure. The most hydrophobic regions are shaded.

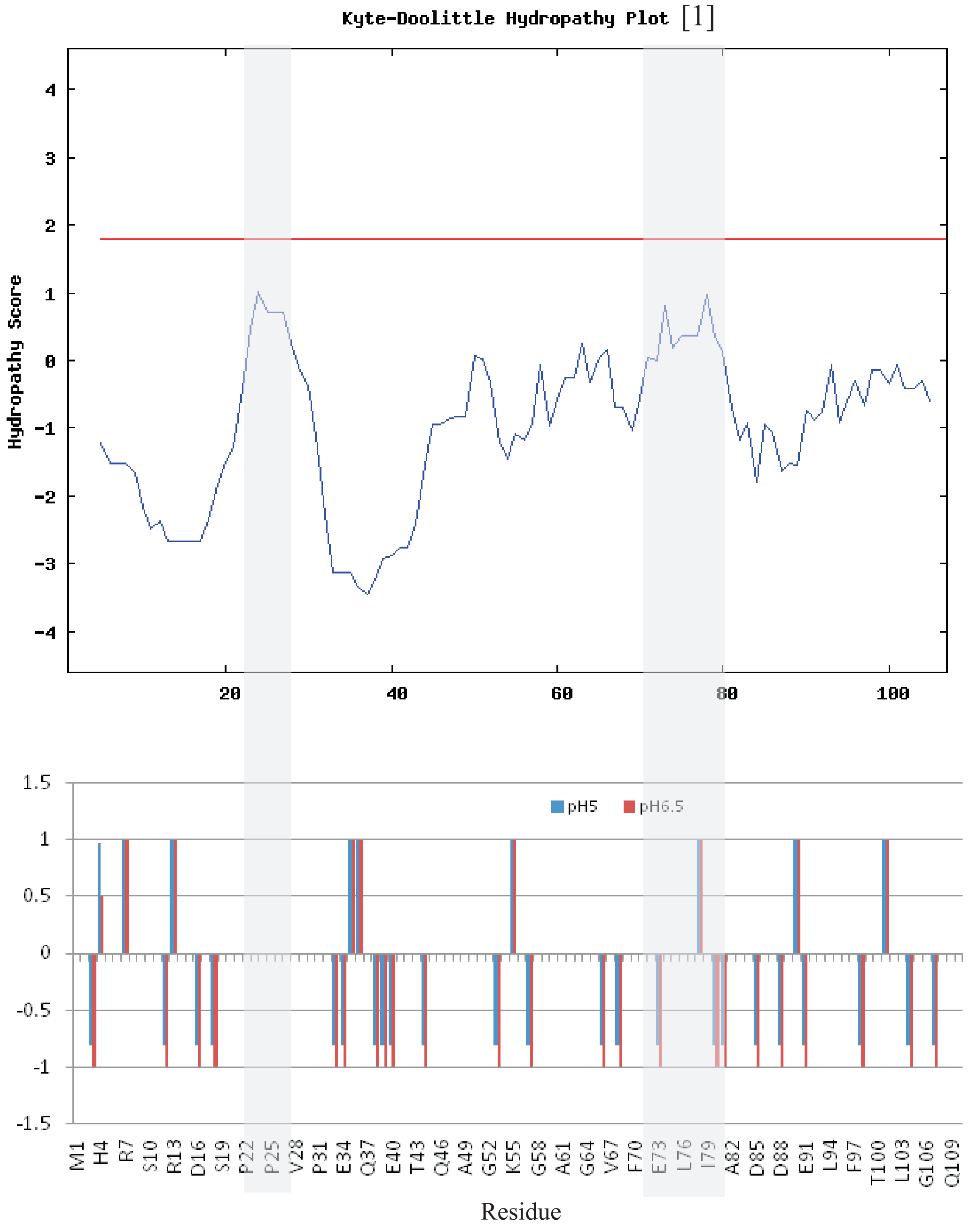

References:
1. Kyte, J. and Doolittle, R. 1982. A simple method for displaying the hydropathic character of a protein. J. Mol. Biol. 157: 105-132.
